# Notes and News

**Published:** 1895-03-02

**Authors:** 


### Notes and News.

Collected and Communicated.

### FORTHCOMING FESTIVALS AND MEETINGS.

The Earl of Rosebery presides at the banquet in aid of the Eveline Hospital, at the Whitehall Rooms, Hôtel Métropole, on March 20th.

His Excellency Count Hatzfeldt will preside at the banquet in aid of the German Society of Benevolence, at the Whitehall Rooms, Hôtel Métropole, on March 30th.

The Duke of Cambridge will preside at the biennial festival of the National Hospital for the Paralysed and Epileptic (Albany Memorial), Queen Square, at the Hôtel Métropole, on April 2nd.

The Hon. W. F. D. Smith, M.P., will preside at the annual dinner of the Newsvendors' Benevolent and Provident Institution, at the Grand Hotel, Trafalgar Square, on April 23rd.

The Duke of Cambridge, President of the Institution, will preside at the 50th anniversary of the German Hospital, Whitehall Rooms, Hôtel Métropole, on April 26th.

Samaritan Free Hospital.—Annual meeting in the Board Room, on March 5th, at 4.30.

New Hospital for Women.—Annual meeting, at the Hospital, on March 11th, at 3.30.

The Hon. W. F. Smith, M.P., will preside at the 82nd anniversary festival of the London Orphan Asylum, Watford, at the Hotel Métropole, on Tuesday, the 26th of March.

The Worshipful Company of Fishmongers have contributed £525 towards the special fund now being raised by the authorities of St. Thomas's Hospital for opening closed wards, and the Directors of the Bank of England have subscribed the same sum.

A useful warning to those who incline towards State-aided hospitals may be drawn from a fact little generally known, which was stated at the meeting in aid of the National Lying-in Hospital, Dublin. It appears that the State, which gave large grants towards the erection of the Rotunda Lying-in Hospital, Dublin, levied a tax on the inhabitants of one particular square in that city, in order to assist in its maintenance. The tax, it seems, still continues, and might certainly form an excuse to the residents in the square for not giving largely in a voluntary manner besides. Another remarkable statement was made at the same meeting. Dr. Boyd said that nearly one-fourth of the population of Dublin were inmates of poor houses, asylums, or jails, whilst another fourth were inmates of charitable institutions, or dependent upon public or private charity, so that there appeared to be one-half of the population completely crippled, whilst the other half was supporting them.

It appears that the medical regulations are not regarded as satisfactory in Belgium, and a Commission has been appointed to inquire into the subject.

The King of the Belgians has sent the Grand Cordon of the Order of Leopold to M. Pasteur, and the Cross of Commander of the same Order to M. Roux.

The Committee of St. George's Hospital have decided to recommend the building of a second operating theatre, at a cost of £10,000; also that a sum not exceeding £18,000 be expended on rebuilding and enlarging the nurses' home. A special inspection of the hospital and of the in-patients by the governors took place yesterday.

At Villuish, in Eastern Siberia, there has just been established a colony of about 40 lepers. These poor creatures at present are housed only under tents, but in the spring huts will be built, and the Government has granted the settlement an annual subsidy, we learn, of 7,000 roubles, a sum sufficient to enable a fuller provision to be made for 100 lepers. Those among the afflicted colony who are able to work engage themselves in gardening, fishing, and hunting. A church is in course of construction for their benefit.

In view of the very strong resolution passed by the General Medical Council regarding the issue of certificates to midwives, which was specially pointed at those given after examination by the Obstetrical Society, Dr. W. S. Playfair has published the exact wording of this celebrated document, which runs as follows: "Obstetrical Society of London. We hereby certify that ............... has passed to our satisfaction the examination instituted by the Obstetrical Society of London, and is, in our opinion, a skilled midwife, competent to attend natural labour." Surely it shows a somewhat vague comprehension of the meaning of the English language that the Medical Council should call this a colourable imitation of a "license to practise medicine, surgery, and midwifery," and should on the strength of it hurl a charge of "infamous conduct" at its most respectable authors.

The first annual report of the National Society for the Employment of Epileptics has been presented to us. Only three months had elapsed when this report was drawn up, since the opening of the colony. There have been none of the ill effects which were apprehended in some quarters, from the association of epileptics in considerable numbers. On the contrary, a general improvement in their physical and mental condition has been very observable. The bracing air of the Buckinghamshire hills, the healthy character of the site on which the colony is situated, and the feeling of comradeship and common interest have worked wonders on the inmates. The charitable public are earnestly urged to assist in extending this humane work to the epileptics scattered amongst us, who may be numbered by tens of thousands. The funds in hand, it is anticipated, will be exhausted in the erection of the new permanent building in course of construction; and, so far, any accommodation for women or children has necessarily been unprovided for until the financial resources are further augmented.

